# Veterinary Medical Society of the University of Pennsylvania

**Published:** 1897-05

**Authors:** John E. Spindler

**Affiliations:** Secretary


Secretary.

### VETERINARY MEDICAL SOCIETY OF THE UNIVERSITY OF PENNSYLVANIA.

The regular bimonthly meeting was held Friday evening, April 2, 1897. Meeting called to order at 8 P.M., President Klein in the chair. Twelve members answered to roll-call. Minutes of previous meeting read and approved.

Committee reported that Dr. Adams would donate the Society the Veterinary Journal for three years back, and keep us supplied with the same in the future. Committee reported that Dr. Harger would donate the Society a copy of *The Exterior of the Horse*. Receipts of the evening were two dollars and ten cents. The Committee on Library Rules reported a set of rules, which, after various amendments, were adopted and the committee discharged with thanks.

The Librarian reported that he had received agricultural reports from Pennsylvania and New York, and also some literature donated by Dr. Hoskins.

Dr. Ravenal was elected an honorary member of the Society.

Mr. Marshall, class of '97, read a very interesting paper on "Rabies." Critic Bower reported.

The programme for the next meeting was read by Mr. Shaw. The meeting adjourned at 10.15 P.M.

The regular bimonthly meeting was held on Friday evening, April 23, 1897. Meeting called to order at 8 P.M., President F. Klein in the chair. Sixteen members answered to roll-call. The minutes of the previous meeting were read and approved.

The Committee on Writing an Article for the *College Annual* reported that no medical society would be allowed to publish an article in that journal. On motion of Mr. Hernshein, the committee was discharged, with thanks. The Committee on Letters of Inquiry made a final report and was discharged, with thanks, on motion of Mr. Beatty.

On motion of Mr. Shaw the regular order of business was then suspended, and Dr. Adams was introduced, who addressed the Society on "Examination for Soundness," giving a demonstration with one of the Department horses.

On motion of Mr. White the thanks of the Society were unanimously tendered the doctor for his kindness.

Mr. Shaw, on behalf of the Executive Committee, then read several estimates for printing new society certificates. On motion of Mr. Beatty the committee was authorized to purchase a better quality of certificate than that at present in use.

Critic Zaner made a favorable report.

The Society adjourned at 11.10 P.M.

JOHN E. SPINDLER,
Secretary.

